# Healthcare Seeking Behavior and Disease Perception Toward Cholera and Acute Diarrhea Among Populations Living in Cholera High-Priority Hotspots in Shashemene, Ethiopia

**DOI:** 10.1093/cid/ciae232

**Published:** 2024-07-12

**Authors:** Tomas Getahun, Dejene Hailu, Ondari D Mogeni, Edlawit Mesfin Getachew, Biruk Yeshitela, Yeonji Jeon, Abel Gedefaw, Samuyel Ayele Abebe, Ermiyas Hundito, David Mukasa, Geun Hyeog Jang, Gi Deok Pak, Deok Ryun Kim, Yeshambel Worku Demlie, Mukemil Hussen, Mekonnen Teferi, Se Eun Park

**Affiliations:** Clinical Trials Directorate, Armauer Hansen Research Institute, Addis Ababa, Ethiopia; Clinical, Assessment, Regulatory, Evaluation (CARE) Unit, International Vaccine Institute, Seoul, Republic of Korea; School of Public Health, Hawassa University, Hawassa, Ethiopia; Clinical, Assessment, Regulatory, Evaluation (CARE) Unit, International Vaccine Institute, Seoul, Republic of Korea; Clinical Trials Directorate, Armauer Hansen Research Institute, Addis Ababa, Ethiopia; Bacterial and Viral Disease Research Directorate, Armauer Hansen Research Institute, Addis Ababa, Ethiopia; Clinical, Assessment, Regulatory, Evaluation (CARE) Unit, International Vaccine Institute, Seoul, Republic of Korea; Clinical, Assessment, Regulatory, Evaluation (CARE) Unit, International Vaccine Institute, Seoul, Republic of Korea; College of Medicine and Health Sciences, Hawassa University, Hawassa, Ethiopia; Statistics and Data Management Department, Armauer Hansen Research Institute, Addis Ababa, Ethiopia; Clinical Trials Directorate, Armauer Hansen Research Institute, Addis Ababa, Ethiopia; Biostatistics and Data Management (BDM) Department, International Vaccine Institute, Seoul, Republic of Korea; Biostatistics and Data Management (BDM) Department, International Vaccine Institute, Seoul, Republic of Korea; Biostatistics and Data Management (BDM) Department, International Vaccine Institute, Seoul, Republic of Korea; Biostatistics and Data Management (BDM) Department, International Vaccine Institute, Seoul, Republic of Korea; Public Health Emergency Management, Ethiopia Public Health Institute, Addis Ababa, Ethiopia; Public Health Emergency Management, Ethiopia Public Health Institute, Addis Ababa, Ethiopia; Clinical Trials Directorate, Armauer Hansen Research Institute, Addis Ababa, Ethiopia; Clinical, Assessment, Regulatory, Evaluation (CARE) Unit, International Vaccine Institute, Seoul, Republic of Korea; Department of Global Health and Disease Control, Yonsei University Graduate School of Public Health, Seoul, Republic of Korea

**Keywords:** healthcare seeking behavior, disease perception, cholera, access to healthcare, Ethiopia

## Abstract

**Background:**

Healthcare seeking behavior (HSB) and community perception on cholera can influence its management. We conducted a cross-sectional survey to generate evidence on cholera associated HSB and disease perception in populations living in cholera hotspots in Ethiopia.

**Methods:**

A total of 870 randomly selected households (HHs) in Shashemene Town (ST) and Shashemene Woreda (SW) participated in our survey in January 2022.

**Results:**

Predominant HHs (91.0%; 792/870) responded “primary health center” as the nearest healthcare facility (HCF). Around 57.4% (247/430) of ST HHs traveled <30 minutes to the nearest HCF. In SW, 60.2% (265/440) of HHs travelled over 30 minutes and 25.9% (114/440) over 4 km. Two-thirds of all HHs paid <USD1 travel cost; SW residents had slightly higher cost burden. When cholera symptoms occur, 68.0% (83/122), 75.5% (114/151), 100.0% (52/52), and 100.0% (426/426) of 0–4, 5–14, 15–17, and ≥18 years, respectively, in ST sought healthcare at our sentinel-HCFs. In SW, younger children visited our sentinel-HCFs slightly more (82.6%, 86.7% in 1–4, 5–14 years, respectively) than older age groups (74.4%, 75.6% in 15–17, ≥ 18 years, respectively). Relatively more adults in ST (12.0%; 51/426) sought over-the-counter drugs at pharmacies than those in SW (2.5%; 11/435). Around 73.8% (642/870) of HHs were aware of cholera disease and 66.7% (428/642) of HHs considered eating unclean food as main causes of cholera.

**Conclusions:**

Variations in cholera prevention practices between rural and urban residents were shown. Addressing differences in HSB per age groups is needed for community engagement for early case detection and case management; critical in reducing cholera deaths and transmission.

## INTRODUCTION

Diarrheal disease is a major global health concern, particularly in developing countries with limited access to water, sanitation, and hygiene (WaSH) [1]. It is the second leading cause of mortality among children under 5 years old resulting in over 525 000 deaths annually worldwide [2]. Sub-Saharan Africa bears a high burden of diarrheal diseases [3], and diarrhea remains a significant public health problem in Ethiopia with prevalence ranging from 19% to 25% among children under 5 years old [4]. The etiology of acute diarrhea includes a wide range of bacterial enteropathogens, parasites, and viruses, many of which are endemic in Ethiopia [1]. Cholera, one of the acute diarrheal diseases, is characterized by frequent outbreaks in Ethiopia. Since 2015, several cholera outbreaks have occurred in different regions of the country [5, 6]. Despite collaborative efforts between the government and stakeholders to control the situation, such as enhancing the disease surveillance, improving WaSH practices, strengthening case detection and management, and vaccination using oral cholera vaccines (OCVs), cholera morbidity and mortality remain significant in cholera endemic and high-priority hotspots like Shashemene areas in the Oromia region [7].

Understanding healthcare seeking behavior (HSB) is crucial for effective cholera control interventions. HSB refers to the actions that individuals take to access healthcare when they are sick [8]. Inadequate HSB contributes to ineffective management of infectious diseases such as cholera and other diarrheal illnesses [8–10]. Delayed HSB, especially for children under 5 years, may lead to preventable deaths [9]. The pooled prevalence of HSB for childhood illness in Ethiopia was 46.6%, increasing the risk of child mortality [11]. Investigating healthcare-seeking patterns within local sociocultural, health system and infrastructural, and accessibility contexts is vital to identify missed opportunities of early cholera case detection and transmission control. Furthermore, how individuals or communities perceive a health problem can have an impact on the management of the disease and how well it is controlled or prevented. Some of these perceptions could stem from cultural practices, religious beliefs, or trust issues. This was evident during the Ebola epidemic of 2014 when it was noted that scientific measures alone were not optimal to control the epidemic without addressing the distinct cultural practices and beliefs of each community or trust issues [12–14]. Some communities align their perceptions with scientific evidence. In the Democratic Republic of the Congo (DRC), a study to examine local perceptions of cholera and vaccine acceptance found that contaminated water and food were considered the main causes of cholera [15], as did another study in 3 African countries—DRC, Kenya, and Zanzibar—including sanitation and dirty environments [16]. Understanding local populations’ disease perceptions is essential for effective cholera management, control, and prevention.

In Ethiopia, although studies have been undertaken in other regions of the country on cholera associated HSB and disease perception, there is paucity of data in Shashemene. The Ethiopia Cholera Control and Prevention Project (ECCP) was instituted in Shashemene in 2021, in contribution to Ethiopia's efforts to implement the national cholera elimination plan (NCP). The overall aim of ECCP was to enhance cholera surveillance, prevent cholera outbreak in a hotspot through a preemptive OCV vaccination campaign and evaluate its impact, and better understand the site-specific cholera associated risk factors. For the latter, a population-based household survey was conducted to assess the cholera-related HSB, WaSH- and socioeconomic-risk factors, vaccination status, and disease perception. Here we present the HSB and disease perception related to cholera and acute diarrhea among populations living in Shashemene, Ethiopia.

## METHODS

### Study Design

This was a cross-sectional household (HH) study conducted in the ECCP surveillance catchment areas in Shashemene Town (ST) and Shashemene Woreda (SW), located in West Arsi Zone in the Oromia regional state of Ethiopia. The survey was implemented during 13–22 January 2022 in ST and 24–30 January 2022 in SW, followed by a mop-up survey to compensate for missing HHs until 14 February 2022. The surveillance catchment population is estimated to be around 325 758, living in 4 kebeles in ST with population size of around 163 546 (Abosto, Alelu, Arada, Awasho) and 4 clusters in SW with population size of around 162 212 (Chebi, Faji Gole, Harabate, Toga). A total of 862 HHs were estimated (426 in ST and 436 in SW) for this survey; and total 870 HHs were interviewed during the actual survey (430 in ST and 440 in SW). HHs were selected using a 2-staged randomization method. For more details on survey methods, please refer to the article by Hailu D and Jeon Y et al [17] included in this *CID* supplement.

### Data Analyses

Data were analyzed using SAS version 9.4 (SAS Inc., Cary, NC, USA). Descriptive statistics for categorical variables related to the type of nearest healthcare facility, mode of transportation, travel distance, travel time, travel cost, and variables related to knowledge and perception were summarized as frequencies and percentages and compared between ST and SW using Pearson Chi-square test and Fisher‘s exact test (expected counts <5). The statistical significance was determined using a *P* value <.05.

## RESULT

### Demographic Characteristics

Overall median age of the respondents was 35 (Q1–Q3: 28–45) years in both ST and SW, and 575 (66.1% of 870) were women; 70.5% (303/430) in ST and 62.0% (272/440) in SW (Table 1). The median HH size was 5 (Q1–Q3: 4–6). Over half of the HHs in ST (54.9%; 236/430) had <5 HH members, whereas more HHs in SW had >5 and <10 HH members (57.4%; 252/440). Nearly 70% of total HH heads interviewed could read (69.2%; 601/870) and write (69.4%; 603/870). HH heads in SW were predominantly farmers (85.4%; 375/440) with primary education (65.8%; 288/440). In comparison, occupation of HH heads in ST were more diversified; 32.3% (139/430) private/self-employment, 23.5% (101/430) laborers, 22.1% (95/430) government employees, 14.7% (63/430) no occupation, and 6.3% (27/430) farmers. More HH heads in ST received secondary (23.3%; 100/430) and tertiary (27.2%; 117/430) education. Overall, 52.5% (229/440) HHs in SW fell into the low wealth index, followed by 29.1% (127/440) in middle wealth index, and 18.3% (80/440) in high wealth index. Comparatively, 41.4% (178/430) of HHs in ST were categorized in high wealth index, followed by 31.9% (137/430) in the low wealth index and 26.7% (115/430) in the middle wealth index. In sum, 42.3% (366/870) of all HHs fell into the lower margin of wealth index.

**Table 1.** Demographic Characteristics of Households in Shashemene Town and Shashemene Woreda

Characteristics	ST Households Surveyed	SW Households Surveyed	Total Households Surveyed	*P*-value
	N = 430 n (% of N)	N = 440 n (% of N)	N = 870 n (% of N)	
**Age of the respondents (years)**				.315
15–24	66 (15.4)	71 (16.2)	137 (15.8)	
25–34	112 (26.1)	127 (28.9)	239 (27.5)	
35–44	136 (31.7)	112 (25.0)	248 (28.6)	
45–54	73 (17.0)	88 (20.0)	161 (18.5)	
≥55	42 (9.8)	41 (9.3)	83 (9.6)	
Median (Q1, Q3)	35 (28, 45)	35 (28, 45)	35 (28, 45)	
**Sex of the respondent**				.008
Male	127 (29.5)	167 (38.0)	294 (33.8)	
Female	303 (70.5)	272 (62.0)	575 (66.1)	
**Household size**				<.001
<5	236 (54.9)	161 (36.7)	397 (45.7)	
5 < 9	183 (42.6)	252 (57.4)	435 (50.1)	
≥10	11 (2.6)	26 (5.9)	37 (4.3)	
Median (Q1, Q3)	4 (3, 6)	5 (4, 7)	5 (4, 6)	
**Age of the household head (years)**				.401
15–24	69 (16.1)	71 (16.2)	140 (16.1)	
25–34	113 (26.4)	126 (28.7)	239 (27.6)	
35–44	134 (31.3)	114 (26.0)	248 (28.6)	
45–54	71 (16.6)	88 (20.0)	159 (18.3)	
≥55	41 (9.6)	40 (9.1)	81 (9.3)	
Median (Q1, Q3)	35 (27, 45)	35 (28, 45)	35 (27.5, 45)	
**Household head can read**				<.001
Yes	324 (75.3)	277 (63.1)	601 (69.2)	
No	103 (24.0)	161 (36.7)	264 (30.4)	
Don’t know	3 (0.7)	1 (0.2)	4 (0.5)	
**Household head can write**				<.001
Yes	325 (75.6)	278 (63.3)	603 (69.4)	
No	103 (24.0)	160 (36.4)	263 (30.3)	
Don’t know	2 (0.5)	1 (0.2)	3 (0.3)	
**Education level of household head**				<.001
Can’t read and write	33 (7.7)	52 (11.9)	85 (9.8)	
Primary	171 (39.8)	288 (65.8)	459 (52.9)	
Secondary	100 (23.3)	73 (16.7)	173 (19.9)	
Tertiary	117 (27.2)	23 (5.3)	140 (16.1)	
Don’t know	5 (1.2)	2 (0.5)	7 (0.8)	
No response	3 (0.7)	0 (0.0)	3 (0.3)	
**Occupation of the household head**				<.001
Farmer	27 (6.3)	375 (85.4)	402 (46.3)	
Laborer	101 (23.5)	21 (4.8)	122 (14.0)	
Government employee	95 (22.1)	13 (3.0)	108 (12.4)	
Private/self-employed	139 (32.3)	25 (5.7)	164 (18.9)	
No occupation	63 (14.7)	3 (0.7)	66 (7.6)	
Others	5 (1.2)	2 (0.5)	7 (0.8)	
**Average monthly income of household head (ETB)**				<.001
≥600 to <1500	4 (1.0)	10 (2.3)	14 (1.6)	
≥1500 to <3000	355 (85.1)	404 (92.2)	759 (88.8)	
≥3000 to <4500	56 (13.4)	23 (5.3)	79 (9.2)	
≥4500 to <6000	2 (0.5)	1 (0.2)	3 (0.4)	
≥6000 to <8000	0 (0.0)	0 (0.0)	0 (0.0)	
≥8000	0 (0.0)	0 (0.0)	0 (0.0)	
**Overall monthly monetary (ETB)**				<.001
≥600 to <1500	123 (28.6)	145 (33.3)	268 (30.9)	
≥1500 to <3000	84 (19.5)	142 (32.6)	226 (26.1)	
≥3000 to <4500	73 (17.0)	64 (14.7)	137 (15.8)	
≥4500 to <6000	68 (15.8)	31 (7.1)	99 (11.4)	
≥6000 to <8000	38 (8.8)	35 (8.0)	73 (8.4)	
≥8000	44 (10.2)	19 (4.4)	63 (7.3)	
**Wealth index of household**				<.001
Low	137 (31.9)	229 (52.5)	366 (42.3)	
Middle	115 (26.7)	127 (29.1)	242 (27.9)	
High	178 (41.4)	80 (18.3)	258 (29.8)	

Abbreviations: ETB, Ethiopian Birr; ST, Shashemene Town; SW, Shashemene Woreda.

### Availability and Accessibility to Healthcare Facility

For the majority of the study participants (792/870; 91.03%), health center was the nearest healthcare facility (HCF) to their HHs in both ST and SW (*P* < .001) (Table 2). Following health center, health post (smaller unit than health center) was the next nearest HCF in SW (229/440; 52.05%) compared to private clinic/hospital in ST (221/430; 51.40%). Local populations in Shashemene predominantly used public transportation to reach HCFs (661/870; 75.98%). The travel time of HHs to the nearest HCF was largely <30 minutes (247/430; 57.44%) or about 30 minutes to <1 hour (174/430; 40.47%) in ST; although over two-thirds of HHs in SW had to travel more than 30 minutes (265/440; 60.23%), nearly 10% (41/440; 9.32%) of households had to travel over 1 hour (1<2 hours), and 2.50% (11/440) of HHs over 2 hours (2<3 hours) to reach the nearest HCF. In terms of travel distance to HCFs, nearly half (210/429; 48.95%) of the respondents in ST traveled between 500 m to 1 km. Comparatively, SW residents had longer travel distance to the nearest HCF with 25.91% (114/440) of HHs at more than 4 km travel distance, followed by 28.18% (124/440) at 2 to < 4 km travel distance. Overall, around two-thirds of HHs in Shashemene paid <USD1 travel cost to HCFs, but residents in rural areas showed slightly higher burden of travel cost to visit HCF (21.36% of SW HHs paid around USD0.53–1.60).

**Table 2.** Availability and Accessibility to Healthcare Facilities Among Study Population in Shashemene Town and Shashemene Woreda

Variables	ST	SW	Total	*P*-value
	N = 430 n (% of N) ± SE	N = 440 n (% of N) ± SE	N = 870 n (% of N) ± SE	
**HCF near to your HH**				
Health post	88 (20.47) ± 6.08	229 (52.05) ± 4.67	317 (36.44) ± 3.83	<.0001
Health center	374 (86.98) ± 2.46	418 (95.00) ± 1.51	792 (91.03) ± 1.44	<.0001
District/rural hospital	101 (23.49) ± 5.97	106 (24.09) ± 5.88	207 (23.79) ± 4.19	.8347
Provincial hospital	33 (7.67) ± 6.55	21 (4.77) ± 6.58	54 (6.21) ± 4.65	.0761
Private clinic/hospital	221 (51.40) ± 4.75	157 (35.68) ± 5.41	378 (43.45) ± 3.61	<.0001
**Mode of transportation**				.0006
Private transportation	33 (7.67) ± 6.55	9 (2.05) ± 6.68	42 (4.83) ± 4.68	
Public transportation	318 (73.95) ± 3.48	343 (77.95) ± 3.17	661 (75.98) ± 2.35	
Walking	77 (17.91) ± 6.18	87 (19.77) ± 6.04	164 (18.85) ± 4.32	
Bicycle	1 (0.23) ± 6.77	1 (0.23) ± 6.77	2 (0.23) ± 4.79	
Other^a^	1 (0.23) ± 6.77	0	1 (0.11) ± 4.69	
Don’t know	0	0	0	
**Travel time**				<.0001
<30 min	247 (57.44) ± 4.45	117 (26.59) ± 5.78	364 (41.84) ± 3.66	
30 to <60 min	174 (40.47) ± 5.26	265 (60.23) ± 4.25	439 (50.46) ± 3.37	
1 to <2 h	6 (1.40) ± 6.78	41 (9.32) ± 6.42	47 (5.40) ± 4.66	
2 to <3 h	2 (0.47) ± 6.84	11 (2.50) ± 6.65	13 (1.49) ± 4.75	
3 to <5 h	1 (0.23) ± 6.77	0	1 (0.11) ± 4.69	
More than 5 h	0	5 (1.14) ± 6.72	5 (0.57) ± 4.76	
Don‘t know	0	1 (0.23) ± 6.77	1 (0.11) ± 4.69	
**Travel cost (ETB [USD])**				<.0001
<5 [0.089]	118 (27.44) ± 5.81	36 (8.18) ± 6.46	154 (17.70) ± 4.35	
5 to <30 [0.53]	283 (65.81) ± 3.99	247 (56.14) ± 4.46	530 (60.92) ± 3.00	
30 to <90 [1.60]	13 (3.02) ± 6.71	94 (21.36) ± 5.98	107 (12.30) ± 4.49	
90 to <160 [2.84]	1 (0.23) ± 6.77	12 (2.73) ± 6.65	13 (1.49) ± 4.75	
160 to <300 [5.33]	0	1 (0.23) ± 6.77	1 (0.11) ± 4.69	
More than 300	0	1 (0.23) ± 6.77	1 (0.11) ± 4.69	
Don‘t know	15 (3.49) ± 6.7	49 (11.14) ± 6.36	64 (7.36) ± 4.62	
**Travel distance**	**N = 429**	**N = 440**	**N = 869**	<.0001
<500 m	59 (13.75) ± 6.34	21 (4.77) ± 6.58	80 (9.21) ± 4.57	
500 m to <1.0 km	210 (48.95) ± 4.88	67 (15.23) ± 6.21	277 (31.88) ± 3.96	
1.0 to <2.0 km	102 (23.78) ± 5.96	103 (23.41) ± 5.90	205 (23.59) ± 4.19	
2.0 to <3.0 km	40 (9.32) ± 6.50	74 (16.82) ± 6.15	114 (13.12) ± 4.47	
3.0 to <4.0 km	12 (2.80) ± 6.73	50 (11.36) ± 6.35	62 (7.13) ± 4.62	
More than 4.0 km	6 (1.40) ± 6.78	114 (25.91) ± 5.81	120 (13.81) ± 4.45	
Don‘t know	0	11 (2.50) ± 6.65	11 (1.27) ± 4.77	

Abbreviations: ETB, Ethiopian Birr; HH, household; HCF, healthcare facility; ST, Shashemene Town; SW, Shashemene Woreda; SE, Standard Error.

The bold values refer to the total number of respondents for this variable (travel distance).

^a^Use cart.

### Age-stratified Healthcare Seeking Behavior for Suspected Cholera

When cholera-like symptoms were experienced, predominant number of respondents preferred to seek healthcare at the ECCP sentinel-HCFs for treatment, followed by the other HCFs both in ST and SW (Table 3). The proportion of respondents who sought healthcare at the ECCP sentinel-HCFs when suspected of cholera increased according to their age in ST: 68.03% (83/122) in children under 5 years of age; 75.50% (114/151) in young children aged between 5 and < 15 years; and 100% (52/52) in adolescents aged between 15 and < 18 years; and 100% (426/426) in adults aged 18 years and above. In comparison, adult population aged 18 years and above in SW showed relatively lower healthcare seeking behavior at the ECCP sentinel-HCFs (329/435; 75.63%). In SW, the children and adolescents went to the ECCP sentinel-HCFs more (0 < 5 years: 82.56% and 5 < 15 years: 86.67%) than the older age groups (15 < 18 years: 74.36% and adults aged 18 years and above: 75.63%). Overall, HHs in both ST and SW preferred to seek healthcare at physician than pharmacy, except for adults in ST. More adults were seeking over-the-counter (OTC) drugs at pharmacies in ST (51/426; 11.97%) than in SW (11/435; 2.53%) and also compared to the other age groups within ST. In SW, less HHs visited pharmacy than physician when any of their HH members showed suspected cholera symptoms across all age groups. Seeking healthcare at traditional healer or self-treatment when suspected with cholera among populations in both SW and ST were minimal.

**Table 3.** Age-stratified Healthcare Seeking Behavior for Suspected Cholera Among Populations in Shashemene Town and Shashemene Woreda

Healthcare Options	Age Groups			
ST (n=325)^a^	0 < 5 y N = 122 n (% of N) ± SE	5 < 15 y N = 151 n (% of N) ± SE	15 < 18 y N = 52 n (% of N) ± SE	Adults^b^ 18 y and above N = 426 n (% of N) ± SE
ECCP-HCF^c^	83 (68.03) ± 7.24	114 (75.50) ± 5.70	52 (100.00) ± 0	426 (100.00) ± 0
Other-HCF^d^	18 (14.75) ± 11.82	28 (18.54) ± 10.39	11 (21.15) ± 17.42	77 (18.08) ± 6.20
Physician	13 (10.66) ± 12.11	5 (3.31) ± 11.31	3 (5.77) ± 19.04	26 (6.10) ± 6.64
Pharmacy	7 (5.74) ± 12.43	6 (3.97) ± 11.27	2 (3.85) ± 19.24	51 (11.97) ± 6.43
Traditional healer	1 (0.82) ± 12.76	0	0	1 (0.23) ± 6.77
Nowhere: self-treatment	0	0	0	0
Nowhere: nothing	0	0	0	0

SW (n=361)^a^	0 < 5 y N = 172 n (% of N) ± SE	5 < 15 y N = 150 n (% of N) ± SE	15 < 18 y N = 39 n (% of N) ± SE	Adults^b^ 18 y and above N = 435 n (% of N) ± SE
ECCP-HCF^c^	142 (82.56) ± 4.50	130 (86.67) ± 4.21	29 (74.36) ± 11.47	329 (75.63) ± 3.34
Other-HCF^d^	23 (13.37) ± 10.03	12 (8.00) ± 11.07	9 (23.08) ± 19.86	59 (13.56) ± 6.30
Physician	6 (3.49) ± 10.59	7 (4.67) ± 11.28	0	34 (7.82) ± 6.51
Pharmacy	1 (0.58) ± 10.74	1 (0.67) ± 11.54	0	11 (2.53) ± 6.70
Traditional healer	0	0	1 (2.56) ± 22.34	0
Nowhere: self-treatment	0	0	0	1 (0.23) ± 6.77
Nowhere: nothing	0	0	0	0

Abbreviations: ECCP, Ethiopia Cholera Control and Prevention Project; HCF, Healthcare Facility; SE, Standard Error; ST, Shashemene Town; SW, Shashemene Woreda.

^a^Total number of households (HHs) with responses for children under 18 years of age was 325 in ST and 361 in SW based on the verified results. The results for the adults are derived from a separate question and not same as that for the children (different denominators).

^b^The results for the adults are derived from a separate question and not same as that for the children.

^c^Healthcare facilities (health centers, hospitals, and private clinics) included in the sentinel HCF-based cholera and diarrhoea disease surveillance under the ECCP project.

^d^Healthcare facilities other than ECCP sentinel HCFs.

### Age-stratified Healthcare Seeking Behavior for Diarrheal Illness

Similar to cholera related healthcare seeking behavior, the majority of HHs sought healthcare predominantly at the ECCP sentinel-HCFs, followed by the other HCFs both in ST and SW, when they experienced diarrheal illnesses (Table 4). Next, a physician was preferred for treatment of diarrhea in both ST and SW, followed by pharmacy. In SW, overall preference for physician was higher than pharmacy across all age groups. The ST populations visited pharmacies more than those living in SW across all age groups. More adults used OTC drugs at pharmacies in ST (20/426; 4.69%) than in SW (2/435; 0.46%) for diarrheal illnesses. Among populations in ST, adults and infants and younger children aged <5 years of age (3/122; 2.46%) showed relatively higher proportion of healthcare seeking at pharmacy. In SW, HHs tend to visit pharmacy when their infants and younger children under 5 years showed diarrheal illness (3/175; 1.71%), whereas the other age groups showed minimal or no reliance on pharmacy. Very few people sought treatment for diarrhea from traditional healers or self-treatment.

**Table 4.** Age-stratified Healthcare Seeking Behavior for Diarrhea Among Populations in Shashemene Town and Shashemene Woreda

Healthcare Options	Age Groups			
ST (n=327)^a^	0 < 5 y N = 122 n (% of N) ± SE	5 < 15 y N = 153 n (% of N) ± SE	15 < 18 y N = 52 n (% of N) ± SE	Adults^b^ 18 y and above N = 426 n (% of N) ± SE
ECCP-HCF^c^	91 (74.59) ± 6.46	122 (79.74) ± 5.15	41 (78.85) ± 9.02	312 (73.24) ± 3.54
Other-HCF^d^	16 (13.11) ± 11.94	23 (15.03) ± 10.54	8 (15.38) ± 18.04	68 (15.96) ± 6.28
Physician	10 (8.20) ± 12.27	6 (3.92) ± 11.21	2 (3.85) ± 19.24	25 (5.87) ± 6.65
Pharmacy	3 (2.46) ± 12.65	2 (1.31) ± 11.37	1 (1.92) ± 19.41	20 (4.69) ± 6.69
Traditional healer	2 (1.64) ± 12.7	0	0	1 (0.23) ± 6.77
Nowhere: self-treatment	0	0	0	0
Nowhere: nothing	0	0	0	0
Don’t know	0	0	0	0

SW (n=364)^a^	0 < 5 y N = 175 n (% of N) ± SE	5 < 15 y N = 149 n (% of N) ± SE	15 < 18 y N = 40 n (% of N) ± SE	Adults^b^ 18 y and above N = 435 n (% of N) ± SE
ECCP-HCF^c^	143 (81.71) ± 4.57	131 (87.92) ± 4.02	29 (72.50) ± 11.72	329 (75.63) ± 3.34
Other-HCF^d^	19 (10.86) ± 10.1	13 (8.72) ± 11.07	6 (15.00) ± 20.61	58 (13.33) ± 6.31
Physician	10 (5.71) ± 10.38	4 (2.68) ± 11.42	4 (10.00) ± 21.21	44 (10.11) ± 6.43
Pharmacy	3 (1.71) ± 10.59	0	0	2 (0.46) ± 6.77
Traditional healer	0	0	1 (2.50) ± 22.08	1 (0.23) ± 6.77
Nowhere: self-treatment	0	1 (0.67) ± 11.54	0	1 (0.23) ± 6.77
Nowhere: nothing	0	0	0	0
Don’t know	0	0	0	0

Abbreviations: ECCP, Ethiopia Cholera Control and Prevention Project; HCF, Healthcare Facility; SE, Standard Error; ST, Shashemene Town; SW, Shashemene Woreda.

^a^Total number of households (HHs) with responses for children under 18 years of age was 327 in ST and 364 in SW based on the verified results. The results for the adults are derived from a separate question and not same as that for the children (different denominators).

^b^The results for the adults are derived from a separate question and not same as that for the children.

^c^Healthcare facilities (health centers, hospitals, and private clinics) included in the sentinel HCF-based cholera and diarrhoea disease surveillance under the Ethiopia Cholera Control and Prevention (ECCP) project.

^d^Healthcare facilities other than ECCP sentinel HCFs.

### Source of Information About Cholera

Overall, healthcare workers (370/642; 57.6%) were found to be the main source of information regarding cholera, followed by community health workers (207/642; 32.2%) and from community meetings (162/642; 25.2%) in Shashemene areas (Table 5). However, when comparing between ST and SW, social media (45/317; 14.2%) and television (137/317; 43.2%) were preferred in ST, although only 5/325 (1.5%) and 15/325 (4.6%), respectively, in SW (*P* < .0001). In SW, community health workers visiting HHs (135/324; 32.9%), community meetings (107/325; 32.2%), and radio (52/325; 16.0%) were more favored (*P* < .0001).

**Table 5.** Knowledge and Perception Toward Cholera by Residents of Shashemene Town and Shashemene Woreda

	ST	SW	Total	*P*-value
	N = 317	N = 325	N = 642	
	n (% of N^a^) ± SE	n (% of N^a^) ± SE	n (% of N^a^) ± SE	
**Source of information on cholera** ^b^				
Family member	56 (17.70) ± 7.21	61 (18.80) ± 7.08	117 (18.20) ± 5.04	.7172
Neighbour/friend	75 (23.70) ± 6.94	69 (21.20) ± 6.96	144 (22.40) ± 4.91	.4608
Healthcare worker	171 (53.90) ± 5.39	199 (61.20) ± 4.89	370 (57.60) ± 3.63	.0617
Radio	22 (6.90) ± 7.64	52 (16.00) ± 7.19	74 (11.50) ± 5.25	.0003
Television	137 (43.20) ± 5.98	15 (4.60) ± 7.65	152 (23.70) ± 4.88	<.0001
Community meeting	55 (17.40) ± 7.23	107 (32.90) ± 6.42	162 (25.20) ± 4.82	<.0001
Community health worker visiting home	72 (22.70) ± 6.98	135 (41.50) ± 6.00	207 (32.20) ± 4.60	<.0001
Social media	45 (14.20) ± 7.36	5 (1.50) ± 7.69	50 (7.80) ± 5.37	<.0001
Other specify^c^	3 (1.00) ± 8.12	0	3 (0.50) ± 5.76	.1198
**Knowledge of measures to prevent cholera** ^d^				
Wash hands with soap and water	193 (60.90) ± 4.97	201 (61.90) ± 4.84	394 (61.40) ± 3.47	.8022
Cook food thoroughly	128 (40.40) ± 6.13	154 (47.40) ± 5.69	282 (43.90) ± 4.18	.0737
Wash vegetables/fruits	124 (39.10) ± 6.19	148 (45.50) ± 5.79	272 (42.40) ± 4.24	.0997
Dispose of human waste properly	93 (29.30) ± 6.68	141 (43.40) ± 5.90	234 (36.50) ± 4.45	.0002
Boil water before drinking	78 (24.60) ± 6.89	90 (27.70) ± 6.67	168 (26.20) ± 4.80	.3737
Clean cooking utensils/vessels	56 (17.70) ± 7.21	37 (11.40) ± 7.39	93 (14.50) ± 5.16	.0238
Treat water with chlorine products	43 (13.60) ± 7.39	30 (9.20) ± 7.46	73 (11.40) ± 5.26	.0837
Cover food to keep away from flies	42 (13.30) ± 7.41	45 (13.90) ± 7.29	87 (13.60) ± 5.20	.8251
Cannot prevent	1 (0.30) ± 7.74	0	1 (0.20) ± 6.32	.4938
**Causes of cholera known by residents**				
Drinking bad/untreated/unboiled	187 (59.00) ± 5.08	200 (61.50) ± 4.86	387 (60.30) ± 3.51	.5095
Eating bad food	202 (63.70) ± 4.79	226 (69.50) ± 4.33	428 (66.70) ± 3.22	.1181
Unwashed fruits/vegetables	149 (47.00) ± 5.78	168 (51.70) ± 5.45	317 (49.40) ± 3.97	.2348
Flies/insects	62 (19.60) ± 7.13	86 (26.50) ± 6.73	148 (23.10) ± 4.90	.0379
Poor hygiene/not washing hands	93 (29.30) ± 6.68	152 (46.80) ± 5.72	245 (38.20) ± 4.39	<.0001
Communal water usage	0	0	0	.4938
Open defecation	0	1 (0.30) ± 7.74	4 (0.60) ± 5.46	.3677
Cultural practices/beliefs	3 (1.00) ± 8.12	1 (0.30) ± 7.74	4 (0.60) ± 5.46	.3677
Other specify^e^	6 (1.90) ± 7.88	0	6 (0.90) ± 5.45	.0141
Don’t know	1 (0.30) ± 7.74	2 (0.60) ± 7.72	3 (0.50) ± 5.76	1.0000
**Knowledge of symptoms associated with cholera**				
Fever	36 (11.40) ± 7.49	16 (4.90) ± 7.63	52 (8.10) ± 5.35	.0028
Vomiting	203 (64.00) ± 4.77	244 (75.10) ± 3.92	447 (69.60) ± 3.08	.0024
Watery diarrhoea	216 (68.10) ± 4.48	244 (75.10) ± 3.92	460 (71.70) ± 2.97	.0512
Stomach/abdominal pain	69 (21.80) ± 7.03	30 (9.20) ± 7.46	99 (15.40) ± 5.13	<.0001
Bloody diarrhoea	32 (10.10) ± 7.53	9 (2.80) ± 7.78	41 (6.40) ± 5.40	.0001
Dehydration	25 (7.90) ± 7.63	6 (1.90) ± 7.88	31 (4.80) ± 5.43	.0004
Other specify^f^	0	1 (0.30) ± 7.74	1 (0.20) ± 6.32	1.0000
Don’t know	6 (1.90) ± 7.88	0	6 (0.90) ± 5.45	.0141

Abbreviations: ST, Shashemene Town; SW, Shashemene Woreda; SE, Standard Error.

^a^The denominator (N) in each column is the number of households (HHs) that responded to the question.

^b^Source of information on cholera: “Religious Leader” and “During OCV vaccination campaign” options with zero responses are not included in this table.

^c^Other specify: “from school” and “tertiary institution”.

^d^Measures to prevent cholera: “Cholera vaccine,” “Other,” and “Don’t know” options with zero responses are not included in this table.

^e^Other specify: Nothing was specified in the responses.

^f^Other specify: “headache” was stated.

### Prevention of Cholera

Regarding knowledge on cholera prevention methods, more than half of the surveyed HHs in both ST and SW (394/642; 61.4%) responded that washing hands with soap and water as the best-known measure to prevent cholera (Table 5). The other known prevention measures were cooking food thoroughly (282/642; 43.9%) and washing fruits and vegetables (272/642; 42.4%). Proper disposal of human waste was favored as a cholera preventive measure by the local populations surveyed in SW (141/325; 43.4%) than in ST (93/317; 29.3%) (*P* = .0002). ST residents were more aware of clean cooking utensils/vessels (56/317; 17.7%) as a measure to prevent cholera as opposed to SW residents (37/325; 11.4%) (*P* = .0238).

### Causes of Cholera

Over two-thirds (428/642; 66.7%) of respondents in ST and SW considered eating unclean food as one of the main causes of cholera, followed by drinking un-boiled/untreated water (387/642; 60.3%) and eating unwashed fruits/vegetables (317/642; 49.4%) (Table 5). Flies/insects (86/325; 26.5%) and poor hygiene/not washing hands (152/325; 46.8%) were also commonly cited as causes of cholera in SW, compared to 62/317 (19.6%) and 93/317 (29.3%) (*p* = .0379 and < .0001), respectively, in ST. Only few residents (4/642: 0.6%) in both SW and ST considered cultural practices/beliefs as causes of cholera.

### Symptoms of Cholera

Most commonly known symptoms to be associated with cholera by HHs were watery diarrhea (460/642; 71.7%) and vomiting (447/642; 69.6%), followed by abdominal pain (99/642; 15.4%) and fever (52/642; 8.1%) in both ST and SW (Table 5). When comparing between ST and SW, there was a slight variability in perception on cholera symptoms by local populations: fever (36/317 [11.4%; *P* = .0028]), stomach/abdominal pain (69/317 [21.8%; *P* < .0001]), bloody diarrhea (32/317 [10.1%; *P* = .0001]), and dehydration (25/317 [7.9%; *P* = .004]) were more commonly identified for ST community, whereas vomiting (244/325 [75.1%; *P* = .0024]) and watery diarrhea (244/525 [75.1%; *P* = .0512]) were more commonly perceived as symptoms of cholera in SW.

### Cholera Disease Treatment Choice and Self-medication

The majority of HHs in Shashemene areas (456/642; 71.0%) opted for going to clinics/hospitals as their primary treatment choice if they had experienced cholera symptoms, followed by oral rehydration solution (ORS) use (159/642; 24.8%) and buying drugs from a pharmacy (37/642; 5.8%) (Table 6). Comparatively, more residents in ST (28/317; 8.8%) purchased drugs from the pharmacy as opposed to those in SW (9/317; 2.8%). Self-medication was found to be uncommon with <10% of HH in both ST and SW answered for this option (582/636; 91.5%).

**Table 6.** Treatment Choices for Suspected Cholera/Diarrheal Illness by Residents of Shashemene Town and Shashemene Woreda

Treatment Choices^a^	ST N = 317 n (% Of N^b^) ± SE	SW N = 325 n (% Of N^b^) ± SE	Total N = 642 n (% of N^b^) ± SE	*P*-value
Go to clinic/hospital	216 (68.10) ± 4.48	240 (73.90) ± 4.01	456 (71.00) ± 3.01	.1110
Use ORS/sugar-salt solution	81 (25.60) ± 6.86	78 (24.00) ± 6.84	159 (24.80) ± 4.84	.6488
Buy drugs from the pharmacy	28 (8.80) ± 7.57	9 (2.80) ± 7.78	37 (5.80) ± 5.44	.0010
Go to a traditional healer	1 (0.30) ± 7.74	2 (0.60) ± 7.72	3 (0.50) ± 5.76	.5774
Home remedy	5 (1.60) ± 7.93	2 (0.60) ± 7.72	7 (1.10) ± 5.57	.2808
Do not treat	1 (0.30) ± 7.74	1 (0.30) ± 7.74	2 (0.30) ± 5.47	1.0000
Other^c^	2 (0.60) ± 7.72	2 (0.60) ± 7.72	4 (0.60) ± 5.46	1.0000

Abbreviations: ORS, Oral Rehydration Solution; ST, Shashemene Town; SW, Shashemene Woreda; SE, Standard Error.

^a^Treatment choices: “Don’t know” option with zero responses not included in this table.

^b^The denominator (N) in each column is the number of households (HHs) that responded to the question.

^c^Other: “Herbal remedies’ and “isolation” were stated.

## DISCUSSION

Our study assessed the accessibility of local populations to HCFs and characterized the HSB including knowledge and perception associated with suspected cholera and other diarrheal illnesses in Ethiopia. In Shashemene area, predominant HHs perceived health centers as the nearest HCF available and accessible, though health posts were more proximal. Local populations had fairly good access to primary HCFs, in terms of physical distance, means of access (public transportation) and cost of travel. A large proportion of respondents sought healthcare predominantly at our ECCP sentinel-HCFs compared to the other healthcare options. The HSB associated with non-cholera suspected diarrheal illnesses was generally similar to the pattern shown for suspected cholera. When cholera symptoms occur, younger children in rural area and older adults in urban setting showed higher proportions of healthcare seeking at our sentinel-HCFs. In ST, younger children under 5 years of age had higher healthcare seeking at pharmacies contrary to the same age group in SW. Healthcare workers were the primary source of information for conveying messages on cholera control and prevention. Over two-thirds of the HHs were aware of cholera disease and considered eating unclean food as main causes of cholera. Respondents recognized watery diarrhea and vomiting to be the main symptoms of cholera. Self-medication was not practiced much, and physicians were considered a preferred option for treatment of acute diarrhea especially among children under 5 years old.

The public health system in Ethiopia has three tiers like most countries. Health posts and health centers are the primary level healthcare providers. Health posts in Ethiopia mainly provide disease preventive services, whereas health center provide comprehensive preventive and curative services. This may explain the high proportion of our study populations seeking healthcare at our sentinel health centers when acute diarrheal symptoms are experienced. Urban populations in Shashemene had relatively better access to health centers as more public and private transportation options were available and shorter travel distance lowered travel costs. To meet the needs of these residents who exhibited high preference of healthcare seeking at health centers, it is important to pre-position cholera rapid diagnostic test (RDT) kits and oral rehydration solution (ORS) [18–21], and strengthen local laboratories and HCF-/lab-personnels. Slight variations in age-group stratified HSB in our study populations showed higher tendency of visiting HCFs in younger children than adults especially in rural areas, like studies in South Africa [22, 23]. In Ethiopia, diarrhea is often perceived by the local communities as self-limiting and thus adults are expected to better withstand the effects compared to children. Further, adults are more likely to have work commitments and other responsibilities that may limit their access to healthcare services in rural populations [24]. Lack of pharmacies in rural areas may have resulted in higher preference towards health centers in these populations, whereas urban populations showed easier access to and potential indiscriminate use of the over-the-counter (OTC) drugs [25–27]. Seeking traditional healers for healthcare for diarrhea appeared very low in Shashemene, contrary to another study in eastern Ethiopia [28]. Healthcare workers being the primary source of information, outreach programs on cholera prevention and control should maximize this channel of community engagement. Several other platforms such as social media, television, and radio could be used appropriately as noted in our survey and found in similar studies in Ethiopia and Haiti [29], [30]. Strong advocacy programs have an impact on cholera control [31], as found in our study. Consumption of unclean food and drinking unboiled water, identified as key cholera risk factors, were consistent with findings of other studies in Ethiopia [32–34]. Despite the good understanding about cholera, outbreaks have been reported in Shashemene. This may be due to the lack of WaSH infrastructures. Around 71.5% (44% in the SW) had access to safe water [17] and hygiene service was the poorest of the WaSH components with only one-third (43.7% in ST and 17.4% in SW) of the HHs reporting basic hygiene services [17].

Our study has several limitations. First, our survey relied on responses of participants without any further verifications such as healthcare records. Second, respondents were HH heads or alternative adults who responded for him/herself and other HH members. Third, the survey conducted in our area may not represent the entire country. However, this is the first study on cholera related HSB conducted in Shashemene area of Ethiopia. Our findings showed the pattern of age-group stratified HSB in urban and rural settings where cholera has been endemic. Active surveillance and community engagement for early case detection of suspected cholera across all age groups are recommended. Health workers and community meetings can be actively utilized to encourage people to visit health centers when experiencing symptoms like acute diarrhea, especially in rural settings. While primary health center was the preferred healthcare seeking option for majority of Shashemene populations, the urban residents exhibited relatively higher OTC purchase compared to rural residents. Close monitoring on the use of antibiotics without proper prescriptions is needed to prevent potential risk of emergence and/or spread of drug resistant cholera, as reported in other parts of Ethiopia [35]. Our data generated evidence on the high knowledge and disease perception of cholera among local populations, but limited WaSH infrastructure was the critical barrier against cholera control. To reduce delayed or inappropriate treatment of cholera patients, timely supply of diagnostic kits and ORS through proper management of inventories at health centers and site laboratories is mandatory.

## CONCLUSION

Understanding HSB on acute diarrhea in cholera endemic and epidemic settings is important for more refined cholera control interventions. HSB in different age groups and urban/rural settings indicate the necessity of tailored approaches. Pre-positioning of diagnostics and ORS supplies on-sites and active community engagement are recommended for early case detection and management. Improvements to WaSH infrastructure and hygiene practices are in dire need.

## Notes

***Author Contributions*.** S. E. P. and M. T. conceptualized the overall study design of the ECCP project. The microplanning of community survey was put together by D. H. in discussion with ECCP team members under the supervision of S. E. P. and M. T. D. H., A. G., B. Y., T. G., and E. M. G. supervised the survey teams during the survey implementation. The survey teams were composed of study RAs and HEWs who conducted household visits and interviews in ST and SW. Y. J., D. H., A. G., O. D. M., T. G., E. M. G., B. Y., and D. M. conducted database review including error checks and corrections. T. G., O. D. M., and E. M. G. drafted the manuscript in discussion with D. H., M. T., and S. E. P. All authors read and provided feedback and approved the final draft.

***Acknowledgments*.** The authors thank the community survey team members including the RAs, HEWs, and local government officials in Shashemene Town and Shashemene Woreda and Oromia Region Health Bureau for their active engagement and support of this survey and ECCP project. They thank the local communities for their consent to take part in the survey. Appreciation is also extended to our research partners and dedicated staff at the ECCP study network in Ethiopia. Thanks to Ms. Bo-Sung Kim and Ms. Hae Kyung Cho for the project operational and administrative support.

***Data sharing*.** All data relevant to the study are included in this manuscript.

***Financial support*.** This study was supported by the Korea Support Committee for International Vaccine Institute (CHMTD05083-010), LG Electronics (CHMTD05083-020), and Community Chest of Korea (CHMTD05083-030). The International Vaccine Institute (IVI) acknowledges its donors, including the government of the Republic of Korea and the Swedish International Development Cooperation Agency.

***Supplement sponsorship.*** This article appears as part of the supplement “Ethiopia Cholera Control and Prevention (ECCP): Evidence-Generation Towards Global Roadmap to Ending Cholera,” sponsored by the IVI.
